# Energy Drink-Associated Electrophysiological and Ischemic Abnormalities: A Narrative Review

**DOI:** 10.3389/fcvm.2021.679105

**Published:** 2021-07-01

**Authors:** Diana X. Cao, Kimberly Maiton, Javed M. Nasir, N. A. Mark Estes, Sachin A. Shah

**Affiliations:** ^1^Department of Pharmacy Practice, Marshall B. Ketchum University College of Pharmacy, Fullerton, CA, United States; ^2^Department of Pharmacy Practice, Thomas J Long School of Pharmacy, University of the Pacific, Stockton, CA, United States; ^3^David Grant USAF Medical Center, Fairfield, CA, United States; ^4^Heart and Vascular Institute, UPMC Presbyterian, Pittsburgh, PA, United States

**Keywords:** energy drinks, electrophysiology, arrhythmia, ischemia, adverse effects

## Abstract

An increasing number of cardiovascular adverse effects, emergency room visits, and deaths have been linked to energy drinks. In this review, we summarized available published literature assessing electrophysiological and ischemic adverse effects associated with energy drink consumption. Overall, 32 case reports and 19 clinical trials are included in this review. Ventricular arrhythmia, supraventricular arrhythmia, and myocardial ischemia were amongst the most commonly reported in case reports with 3 having a fatal outcome. Although serious ischemic changes, arrhythmias, or death were not observed in clinical trials, significant electrophysiological changes, such as PR/PQ interval shortening/prolongation, QT/QTc shortening/prolongation, and ST-T changes, were noted. QT/QTc interval prolongation appears to be the most significant finding in clinical trials, and there appears to be a dose-response relationship between energy drink consumption and QTc prolongation. The exact mechanisms and the particular combination of ingredients behind energy drink-induced cardiac abnormalities require further evaluation. Until more information is available, energy drink use should be considered as part of the differential diagnosis in appropriate patients presenting with electrocardiographic changes. Further, certain patient populations should exercise caution and limit their energy drink consumption.

## Introduction

Energy drinks (ED) are an increasingly utilized niche in the beverage market and are gaining popularity in recent years, especially amongst teenagers and young adults. According to the National Health and Nutrition Examination Surveys, ED consumption has grown substantially amongst adolescents and adults in the United States (1). A United States Substance Abuse Services and Mental Health Administration (SAMHSA) report revealed patients aged 18 to 39 were most commonly involved in ED-related emergency department visits (2). Energy drinks are generally available in a variety of sizes ranging from a 60 ml “shot” form, to a larger 710 ml container. They typically contain caffeine in conjunction with presumed energy-enhancing ingredients such as taurine, B vitamins, and herbal extracts. Caffeine content in ED typically vary from 70 to 200 mg per 473 ml serving (3). Beneficial claims of ED include improvements in alertness, physical endurance, metabolism and concentration. Energy drink-associated emergency department visits have sharply increased in recent years. According to a report by SAMHSA, the number of emergency department visits due to EDs increased from 10,068 to 20,783 between 2007 and 2011 (2). In a recent study conducted in Air Force personnel, nearly 1% of energy drink users reported needing to see a physician or going to the emergency department because of adverse effects from energy drinks (4). The Center for Science in the Public Interest reports 34 deaths linked to energy drink consumption (5). An analysis of poison control data of 5,156 reports suggests cardiac and neurological adverse effects are amongst the most common (6). It is important to note, however, that the number of adverse events due to ED is likely underestimated in these reports since such reporting is completely voluntary. Accordingly, we reviewed available literature of published case reports and clinical research studies evaluating electrophysiological abnormalities related to ED consumption. This review serves to better inform clinicians of the notable connection between ED and electrocardiogram (ECG) changes. The information may provide additional insight in the differential diagnosis of patients presenting with ECG changes in the emergency department.

## Methods

A qualitative review was conducted by searching for relevant articles in PubMed, EMBASE, Cochrane database from 2001 to July 1, 2019 along with hand searching references from appropriate articles. Case reports and clinical trials assessing the effects of energy drink on electrophysiological and ischemic abnormalities were included. Studies assessing energy capsule, bar, soda or sports drink were excluded. Due to the sparsity of randomized, placebo-controlled trials and the heterogeneity among these studies, a meta-analysis was not performed.

## Results

A total of 32 individual cases (28 case report publications) have been described in the literature regarding ED and cardiac rhythm abnormalities (Table 1) (7–35). Three cases were described in two publications by Mattioli et al. and each case was only counted once as the authors assumed that the two publications were referring to the same patients. The majority of patients were male (84%) with a median age of 25 years (range 13–53). Seventeen cases were from the United States. The most common presentations were chest pain, palpitation, nausea/vomiting, and cardiac arrest. Twenty-two cases involved consumption of ED only, while 10 reported coingestion of an ED with another substance (5 with alcohol, 3 with amphetamine salts, 1 with additional caffeine, and 1 with a caffeinated soda). A total of 3 cases resulted in death, with one of the cases reporting coingestion with 3, 4-methylenedioxymethamphetamine (MDMA). Arrhythmia was reported in 20 cases (9 supraventricular [includes one case of Wolff-Parkinson-White Syndrome], 10 ventricular and 1 being supraventricular and ventricular). Fourteen cases reported ST-segment changes, 1 of which was fatal, 5 were found to have coronary artery occlusion, 2 were diagnosed with spontaneous coronary artery dissection, 1 had a normal repeat exercise treadmill test without ED, 4 revealed normal coronary arteries post coronary angiography, 1 was diagnosed with Brugada syndrome, and 1 was diagnosed with coronary artery vasospasm. QT/QTc prolongation was evident in 4 cases. One case was associated with ventricular tachycardia, 2 were discharge with a diagnosis of Long QT type 1 (LQTS1), and QTc prolongation resolved without intervention in 1 case involving a healthy volunteer. One patient was identified as having reverse Takotsubo cardiomyopathy.

**Table 1:** Case reports of energy drinks and related electrophysiological and ischemic abnormalities (7–35).

**Author, Year (Country)**	**Presentation**	**Age/Gender**	**Energy drink & co-ingestions (quantity)**	**Electrophysiological abnormalities identified**	**Outcomes**
Zacher et al., 2018 (35) (Germany)	Severe persisting chest pain	25/M	Various unstated caffeinated energy drinks (~2 l) mixed with strong liquor	ST-segment elevation in leads I, aVL and V1–V4 with corresponding inferior ST-segment depression, as well as in V6 and the dorsal leads	Spontaneous LAD dissection and concomitant occlusion; Drug eluting stent placement with restoration of TIMI 3 flow
Ullah et al., 2018 (32) (United States)	Substernal chest pain, shortness of breath, nausea, and vomiting	25/M	Unstated caffeinated energy drinks (7–9 cans) daily for the past week	ST depression in precordial leads V2–V6	Normal coronary arteries; symptom free on follow-up
Demir et al., 2018 (12) (Turkey)	Tachycardia and feeling of a burning sensation in the chest	34/M	Red bull (2 boxes)	Wide QRS tachycardia with delta waves	Converted to NSR; radiofrequency ablation of left posterolateral accessory pathway after diagnosis of intermittent WPW syndrome; drug eluting stent placement
Choudhury et al., 2017 (11) (United Kingdom)	Abnormal exercise treadmill test	53/M	Red bull (2 cans)	PVCs and ST changes during exercise treadmill test	Normal exercise treadmill test on repeat testing (with no ED) one week later
Enriquez and Frankel, 2017 (15) (United States)	Cardiac arrest	19/M	Monster (3 cans of 8 oz)	VF	Converted to NSR; full neurologic recovery
	Syncope	23/F	Red bull (1 can)	VF	Appropriate shock by AICD; successful conversion to NSR
Hernandez et al., 2016 (17) (United States)	Acute crushing retrosternal chest pressure and shortness of breath	28/M	Red Bull (4–5 drinks daily × several weeks)	Initial ECG: hyper-acute T waves in leads II, III, aVF, and ST-segment depression in leads I, aVL, V1, and V2 Repeat ECG: 3 mm ST segment elevation with tombstone morphology in leads II, III, aVF, and ST segment depression in I and aVL	Complete occlusion of mid-RCA; aspiration thrombectomy with TIMI 3 flow
Mattioli et al., 2016 and 2018 (20, 21) (Italy)	Precordial oppressive sensation, palpitations, increasing anxiety, and nausea	22/M	Unstated caffeinated energy drink (750 ml)	AF with ventricular rate of 135 bmp	Converted to NSR
	Palpitations and anxiety	23/M	Unstated caffeinated energy drink (600 ml) with recent increase in caffeine intake (400 mg/day)	AF with ventricular rate of 150 bmp	Converted to NSR
	Anxiety, nausea, increasing precordial discomfort, and palpitations	26/M	Unstated caffeinated energy drink (600 ml) with alcoholic beverage (corresponds to 30 g of alcohol)	AF with ventricular rate of 170 bmp	Converted to NSR
Sattari et al., 2016 (27) (United States)	Bloody vomit	28/M	Monster (2 drinks daily × several months) and beer (2–3 drinks daily × several months)	AF with ventricular rate of 130 bpm	Converted to NSR
Khan et al., 2015 (19) (United States)	Unresponsive, pulseless	27/M	Red Bull (6 drinks daily ×6–8 months)	Initial ECG: VT ECG after shock: non-specific ST-T wave changes; QTc 469 ms Repeat ECG: borderline lateral ST elevation	Converted to NSR; normal coronary arteries
Solomin et al., 2015 (30) (United States)	Left-sided chest pain	26/M	Monster, Rock Star, and other similar brands of energy drink (~4 L/day, duration unspecified)	ST elevation in inferior leads with reciprocal changes in anterior leads	Complete occlusion of left circumflex artery; drug-eluting stent placement with resolution of ST elevation
Unal et al., 2015 (31) (Turkey)	Retrosternal chest pain, palpitations, and emesis	32/M	Unstated energy drink (5 bottles)	ST elevation V2 through V6	Left main and proximal LAD thrombus; no atherosclerotic lesions or coronary malformations; LAD balloon angioplasty with proximal LAD flow; discharged home with DAPT
Shah et al., 2014 (29) (United States)	QTc prolongation	31/M	Phase A: Monster (32 oz) Phase B: carbonated water (16 oz) twice daily ×7 days (total daily volume was consumed together for one of the 7 days) followed by Monster (16 oz) twice daily ×7 days	Phase A: maximum observed change in QTc = 25 ms Phase B: maximum post-dosing change in QTc from placebo QTc = 22 ms; no VT, SVT, AF, or AV block	QTc prolongation resolved without intervention
Ward et al., 2014 (33) (United States)	First AICD shock	45/M	Red bull (3 drinks)	Non-sustained VT/VF	Fatal arrhythmia prevented by AICD shock
Avci et al., 2013 (7) (Turkey)	Loss of consciousness/cardiac arrest	28/M	Unstated caffeinated energy drink (750 ml, 250 ml daily ×7 months)	VT	Converted to NSR, followed by SCD 3 days later
Polat et al., 2013 (24) (Turkey)	Crushing, mid-sternal chest pain	13/M	Unstated caffeinated energy drink (volume unstated)	2 to 3 mm ST segment elevation in leads II, III, aVF, and V3–V5	Spontaneous LAD dissection with visible tear; discharged home after medical treatment
Benjo et al., 2012 (8) (United States)	Nausea, multiple episodes of emesis, palpitations, and severe retrosternal chest pain	24/M	Unstated energy drink mixed with vodka (3 drinks)	Initial ECG: NSR with subtle J-point elevation in leads II, III, aVF, and V2–V6 with a concave shape Repeat ECG: ST elevation and a convex shape in the lateral leads, with a decrease in R-wave progression	Left main (involving the origin of the circumflex) and LAD thrombus with no atherosclerotic lesions or coronary malformations; Discharged home after CABG
Dufendach et al., 2012 (14) (United States)	Palpitations, chest pain, shakiness, dizziness	13/F	Unstated caffeinated energy drink (≥16 oz every other day ×2 weeks)	QT/QTc = 420/561 ms	Diagnosis of LQT1; normal QTc
Hanan Israelit et al., 2012 (16) (Israel)	Crushing chest pain, nausea, vomiting	24/M	Unstated caffeinated energy drink (XL, 20 cans); MDMA	Initial ECG: widespread ST segment elevation While waiting for catheterization: wide complex tachycardia, which further deteriorated to VF	Death
Kaoukis et al., 2012 (18) (Greece)	Chest pain, acute respiratory failure, palpitations	24/M	Unstated caffeinated energy drink (volume unstated)	SVT; VT; sinus tachycardia; mildly elevated troponin; LVEF = 35%; elevated BNP	Diagnosis of reverse takotsubo cardiomyopathy
Mugmon (2012) (United States)	Lightheadedness and palpitations	26/M	5-h energy drink (138 mg caffeine, volume unknown) with mixed amphetamine salts	Atrial flutter with 1:1 conduction and aberrant conduction (300 bpm); AF and other atrial arrhythmias	Successful ablation
Rottlaender et al., 2012 (25) (Germany)	Cardiac arrest	22/F	Unstated caffeinated [480 mg] energy drink (6 cans)	TdP degenerated to VF; QT/QTc = 526/492 ms	Diagnosis of LQT1; normal QTc; no coronary anomalies
Rutledge et al., 2012 (26) (United States)	Collapse	24/M	Red bull with vodka (a few sips)	VF; R' with ST segment elevation in V1 and V2	Converted to NSR; diagnosis of Brugada syndrome; discharged with AICD
Wilson et al., 2012 (34) (United States)	Acute chest pain	17/M	Red Bull (3–4 cans) and Monster (2–3 cans)	Diffuse ST elevation in leads II, III, AVF, V3–V6, and ST segment depression in leads AVR and V1	Spontaneous normalization of ECG; diagnosis of acute coronary artery vasospasm
Di Rocco et al., 2011 (13) (United States)	Persistent heart fluttering	14/M	Unstated caffeinated drink day prior (volume unknown)	AF with occasional atrial flutter	Converted to NSR
	Intoxication and vomiting	16/M	Red Bull with vodka (volumes unknown); amphetamine and dextroamphetamine 30 mg/day at home	Atrial tachycardia; AF with RVR	Converted to NSR
Scott et al., 2011 (28) (United Kingdom)	Acute chest pain	19/M	Red Bull (2–3 cans daily ×1 week)	2 mm ST segment elevation in leads I, II, aVL and V4–V6, with 2 mm ST depression in leads V1 and V2	Normal coronary arteries
Berger and Alford, 2009 (9) (Australia)	Cardiac arrest	28/M	Unstated caffeinated energy drink (7–8 cans)	Initial ECG: VF ECG on arrival at hospital: elevated anteroseptal ST segments with reciprocal inferior ST depression	Converted to NSR; normal coronary arteries
Nagajothi et al., 2008 (23) (United States)	Palpitations and chest tightness	23/F	GNC Speed Shot; Mountain Dew soda (volumes unstated)	Narrow complex tachycardia	Converted to NSR
Cannon et al., 2001 (10) (Australia)	Collapse	25/F	Race 2005 Energy Blast (55 ml)	VF	Death

*AF, atrial fibrillation; AICD, automated implantable cardioverter defibrillator; AV, atrioventricular; bpm, beats per minute; CABG, coronary artery bypass grafting; DAPT, dual antiplatelet therapy; ECG, electrocardiogram; ED, energy drink; LAD, left anterior descending; LQTS1, long QT type 1; MDMA, 3,4-methylenedioxymethamphetamine; NSR, normal sinus rhythm; PVC, premature ventricular contraction; RCA, right coronary artery; RVR, rapid ventricular response; SCD, sudden cardiac death; ST, sinus tachycardia; STEMI, ST-elevation myocardial infarction; SVT, supraventricular arrhythmia; TdP, torsades de pointes; VF, ventricular fibrillation; VT, ventricular tachycardia; WPW, Wolff-Parkinson-White*.

Table 2 contains a list of all 19 original research articles (36–54). The smallest sample size studied was 1 (this study was designed as a clinical trial but stopped early due to administrative reasons and the emergence of new data), and the largest study included 80 participants. Subjects were generally young healthy volunteers with the exception of the study by Gray et al. which evaluated subjects with long QT syndrome (LQTS). Caffeinated EDs were used in all studies. The volume of EDs or matching control drinks ranged from 60 to 1,000 ml. The shortest total duration of assessment was 30 min, while the longest lasted 24 h. Ten out of the 19 studies were performed in the United States. Thirteen were controlled trials, whereas 6 were non-controlled. Three studies employed a parallel study design, 8 employed crossover, and 1 study used both parallel and crossover design. All trials assessed the effect of ED on QTc interval. In 8 of the 19 studies, ED was associated with statistically significant QTc prolongation compared to at least one comparator group. In contrast, 1 study showed ED-associated QTc shortening. QT interval correction were most commonly calculated using the Bazett's formula (9 out of 19 studies). Of the remaining studies, 3 used both Bazett's and Fridericia formula, and 7 did not specify the formula used for QTc correction. Eighteen of the trials evaluated the effect of ED on heart rate (HR). Of these studies, 5 demonstrated a statistically significant increase in HR with ED in at least one comparison, and 1 found a statistically significant HR decline. ED was associated with more frequent ST-T changes in 1 study, and none of the studies noted any clinically significant arrhythmias.

**Table 2:** Clinical trials of energy drinks and related electrophysiological and ischemic abnormalities (36–54).

**Author, Year (Country)**	Patient population, age^*^	**Study design**	**Sample size**	**Energy drink (volume)**	**Control drink (volume)**	**Assessment duration**	**Electrophysiological endpoints^†^**	**Outcomes**
Basrai et al., 2019 (38) (Germany)	Healthy, 18–25 y	R, DB, C, P, & CO	38	Red Bull (750 ml, 1,000 ml)	Non-caffeinated control drink (750, 1,000 ml) [CP] Non-caffeinated control drink with taurine (75, 1,000 ml) [CP + T] Caffeinated control drink (750, 1,000 ml) [CP + C] Caffeinated control drink with taurine (1,000 ml) [CP + C + T] Non-caffeinated control drink with glucuronolactone (1,000 ml) [CP + G]	11 h	BP, HR, QTc	QTc prolongation at 1 h compared to control [8 ± 17 ms vs. 2 ± 15 ms (CP), 1 ± 16 ms (CP + C), −3 ± 17 ms (CP + T), −9 ± 16 ms (CP + C + T), and −9 ± 19 ms (CP + G), *P* = 0.0021] QTc shortened after 1 h compared to the baseline in the CP + C + T and CP + G group (*p* < 0.05) HR increased at 1 h compared to control [3.5 vs. 0 bpm (CP), 0 bpm (CP + C), 1.0 bpm (CP + T), −1.0 bpm (CP + C + T), and −1.0 bpm (CP + G), *p* = 0.017] No arrhythmias
Shah et al., 2019 (51) (United States)	Healthy, 22 ± 3 y	R, DB, PC, CO	34	Unstated caffeinated energy drink A (32 oz) Unstated caffeinated energy drink B (32 oz)	Non-caffeinated control drink (32 oz)	240 min	QTcB, QTcF, QT, PR, QRS, HR	Maximum change from baseline in QTcB for Drink A and Drink B were higher than placebo (18 ± 14 ms, 20 ± 16 ms, and 12 ± 11 ms, for Drink A, Drink B, and placebo, respectively, *p* = 0.005) Maximum change from baseline in QTcF for Drink A and Drink B were higher than placebo (15 ± 12 ms, 15 ± 12 ms, and 7 ± 7 ms, for Drink A, Drink B, and placebo, respectively, *p* < 0.001) Two participants had a change from baseline in QTcB of >50 ms with Drink A and Drink B No significant change in PR, QRS, and HR
McGaughey et al., 2018 (46) (United States)	Heathy, 26 y	R, DB, C, CO	1	Unstated caffeinated energy drink (32 oz) with placebo capsule	Placebo drink (32 oz) with moxifloxacin 400 mg ×1 Placebo drink (32 oz) with placebo capsule	6 h	QTcB, QTcF, QT, PR, QRS, HR	Maximum baseline-adjusted, placebo-corrected, change in QTcB was 29 and 12 ms with energy drink and moxifloxacin, respectively
Brothers et al., 2017 (39) (United States)	Healthy, 27 ± 4 y	R, DB, PC, CO	15 for each protocol	Protocol 1: Monster (2 mg caffeine/kg) Monster (3 mg caffeine/kg) Protocol 2: Monster (16 oz) Monster (24 oz)	Protocol 1: Keurig K-cup (2 mg caffeine/kg) Water (250 ml) Protocol 2: Keurig K-cup (1 packet) Volume unknown Water (250 ml)	6.5 h	QTcB, HR	No statistically significant difference in QTcB and HR in both protocols
Fletcher et al., 2017 (41) (United States)	Healthy, 27 ± 4 y	R, DB, C, CO	18	Unstated caffeinated energy drink (946 ml)	Caffeine-matched control drink (946 ml)	24 h	QTc, QT, PR, QRS, HR	QTc prolongation 2 hrs after ED consumption when compared to caffeine control (0 ± 18 ms vs. −10 ± 15 ms; *p* = 0.02) No statistically significant difference in QT, PR, QRS and HR
Garcia et al., 2017 (42) (Columbia)	Healthy, 21 ± 2 y	R, DB, P, PC	80	Unstated caffeinated drink A (460 ml) Unstated caffeinated drink B (460 ml) Unstated caffeinated drink C (460 ml)	Carbonated water (460 ml)	1 h	QTc, PR, QRS, HR, T wave amplitude	Shortening of QTc in group B after drink intake Percent change in QTc not different in any groups compared to control No other significant ECG parameter changes Percent increase in HR was significant in A and C groups compared to control (*p* = statistically significant)
Gray et al., 2017 (43) (Australia)	LQTS, 29 ± 9 y	R, DB, PC, CO	24	Red Bull (500 ml)	Non-caffeinated control drink (500 ml)	90 min	QTcB, HR	No statistically significant change in QTcB and HR 3 patients had QTcB changes ≥50 ms No arrhythmias
Tauseef et al., 2017 (53) (Pakistan)	Healthy, 19–21 y	R, C, P	30	Red Bull (500 ml) [High dose] Red Bull (250 ml) [Low dose]	No drink	2 h	QTc, HR	Statistically significant increase in QTc at 2 h: High dose ED vs. low dose ED (difference of 9 ± 3 ms; *p* = 0.038); high dose ED vs. control (difference of 13 ± 3 ms; *p* = 0.002) Statistically significant increase in HR at 1 and 2 h with high dose ED when compared to other groups (*p* ≤ 0.015 for all comparisons)
Hajsadeghi et al., 2016 (44) (Iran)	Healthy, 23 ± 4 y	OL, NC	44	Unstated caffeinated energy drink (250 ml)	N/A	4 h	QTc, PR, QRS, ST-T changes, HR	No changes in PR, QRS, and QTc from baseline More frequent ST-T changes from baseline (*p* = 0.004) Significant reduction in HR at all times compared to baseline (*p* ≤ 0.043 for all comparisons)
Kozik et al., 2016 (45) (United States)	Healthy, 29 y	OL, NC	14	Monster (946 ml)	N/A	4 h	QTcB, HR	Significant increase in maximum QTcB interval (baseline = 423 ± 23; post ED = 503 ± 25; *p* < 0.001) Maximum QTcB prolongation >500 ms: 8/14 subjects Maximum QTcB prolongation >50 ms from baseline: 8/12 subjects (missing baseline QTcB in 2 subjects) T-wave changes: 9/14 subjects
Shah et al., 2016 (48) (United States)	Healthy, 38 y	OL, C	24	5-H Energy (60 ml daily)	No drink	2 h (with exercise ECG stress test)	QTc, PR, QRS, HR, arrhythmias	QTc prolonged significantly before and after ED (412 to 434 ms; *p* ≤ 0.001) No change in PR, QRS, or HR No arrhythmias
Shah et al., 2016 (49) (United States)	Healthy, 22 ± 3 y	R, DB, PC, CO	27	Unstated caffeinated energy drink (946 ml)	Non-caffeinated control drink (946 ml) Non-caffeinated control drink with Panax Ginseng (946 ml)	5.5 h	QTcB, PR interval, QRS duration, QT, HR	QTcB prolongation 2 h after ED consumption when compared to placebo-control (3 vs. −3 ms respectively; *p* = 0.03) PR interval significantly decreased after ED consumption compared to placebo at 1 h and ginseng control at 1 and 2 h (*p* ≤ 0.025 for all comparisons) No significant changes in QRS, QT, HR.
Shah et al., 2016 (49) (United States)	Healthy, 28 ± 6 y	R, DB, PC, CO	26	5-H Energy (60 ml twice daily for 7 days)	Non-caffeinated control drink (60 ml)	5 h on days 1 and 7	QTcB, QT, PR, QRS, HR	No significant change in QTcB, PR, QRS, and HR after single ED shot or chronic consumption
Alsunni et al., 2015 (36) (Saudi Arabia)	Healthy, overweight/obese, 21 ± 1 y	OL, NC	31	Unstated caffeinated energy drink (5 ml/kg)	N/A	1 h	QTcB, ECG, HR variability	QTcB significantly increased in the overweight/obese group after ED consumption (357 ± 54 ms to 340 ± 57 ms, *p* = 0.006) No changes in QTcB in the normal weight group HR variability (as indicated by the expiratory to inspiratory ratio, the mean heart rate range, and the mean percentage variability) was significantly less in overweight/obese as compared with normal weight after ED consumption (*p* ≤ 0.04 for all comparisons)
Elitok et al., 2015 (40) (Turkey)	Healthy, 25 ± 2 y	OL, NC	50	Red bull (355 ml)	N/A	2 h	QTcB, Tp-e interval, Tp-e/QTc ratio, PR, QRS, HR	No significant change with QTcB, PR, QRS, Tp-e related parameters after ED consumption HR significantly increased after ED consumption at 1 h (78 ± 15 to 84 ± 12 bmp, *p* = 0.008) and 2 h (78 ± 15 to 85 ± 11 bmp, *p* = 0.005)
Arinc et al., 2013 (37) (Turkey)	Healthy, >17 y	OL, NC	20	Red bull (250 ml)	N/A	2 h	QTcB, p-wave dispersion, QT dispersion, HR	No significant changes in QTcB, p-wave dispersion, QT dispersion, and HR after ED consumption
Ragsdale et al., 2010 (47) (United States)	Healthy, 20 ± 2 y	DB, PC, P	68	Red bull (250 ml, normal calorie) Red bull (250 ml, low calorie)	Non-caffeinated control drink (250 ml, normal calorie) Non-caffeinated control drink (250 ml, low calorie)	2 h	ECG (QTc, QT), consecutive NSR that exceed 50 ms, % RR with intervals > 50 ms, QRS, ST, count of t-wave inversion, HR, HR variation	No significant change in QTc, QRS, ST, T-wave inversion count, and HR
Steinke et al., 2009 (52) (United States)	Healthy, 26 ± 6 y	OL, NC	15	Unstated caffeinated energy drink (500 ml daily ×7 days)	N/A	4 h on days 1 and 7	ECG parameters (QTcB & QTcF), HR	No significant change in QTcB and QTcF with ED consumption on days 1 and 7 ~QTcB trending toward significance on day 7 (20 ms, *p* = 0.052) HR increased significantly after ED consumption on days 1(7.8%, *p* = 0.009) and 7 (11%, *p* < 0.001)
Wiklund et al., 2009 (54) (Sweden)	Healthy, 19–30 y	OL, C, CO	10	Red bull (750 ml) Red bull (750 ml with 0.4 g of ethanol/kg)	No drink	30 min of exercise	Changes in ECG (QTcB, PQ, QRS), HR, HR variability and recovery	No significant change in QTcB and QRS after ED consumption (alone or in combination with ethanol) Significant increase in PQ after ED consumption and before exercise (alone or in combination with ethanol, *p* < 0.05) No significant change in HR, but HR recovery and variability slower with ED and ethanol No arrhythmias

*R, randomized; DB, double-blind; C, controlled; P, parallel; CO, cross over; PC, placebo controlled; OL, open label; NC, non-controlled; bpm, beats per minute; ECG, electrocardiogram; ED, energy drink; HR, heart rate; LQTS, Long QT syndrome; NSR, normal sinus rhythm; O, obese; OW, overweight; Tp-e, interval from the peak to the end of the electrocardiographic T wave*.

* *Age is reported as mean; mean ± standard deviation; or range*.

† *QTcB, Corrected with Bazzett formula; QTcF, Corrected with Fridericia formula; QTc, Correction formula unknown*.

## Discussion

In our comprehensive review of case reports with a documented electrophysiological abnormality, ventricular arrhythmia, supraventricular arrhythmia, and myocardial ischemia were amongst the most commonly reported. Although serious arrhythmias or death were not observed in clinical trials, a multitude of significant ECG changes (PR/PQ interval shortening/prolongation, QT/QTc shortening/prolongation, ST-T changes) with ED were noted. QTc prolongation was the most common and significant finding in clinical trials. In controlled trials showing a significant prolongation of the QT interval, the average change in QT/QTc was small (6–10 ms) (38, 41, 50, 51), but this magnitude of change can still be meaningful. The FDA requires thorough QT/QTc evaluation for all new drug entities with a QT/QTc prolongation of at least 5 ms (55), and results of such evaluation may carry drug labeling significance. As an example, azithromycin bears a warning in its package insert for prolonging the QTc by 5–9 ms with the administration of 500–1,500 mg of oral azithromycin (56). QT/QTc changes >50 ms or an absolute value >500 ms are generally considered clinically significant in practice (55), and this degree of QT/QTc prolongation was observed in a few cases and several clinical trials participants (14, 25, 43, 45, 51).

Our review identified 3 cases resulting in death, all of which had a documented ventricular arrhythmia prior to the fatal event. Sudden cardiac death in patients under the age of 35 is usually attributed to an underlying cardiac condition but is also frequently idiopathic (57); however, the temporal relationship between energy drink consumption, ventricular arrhythmia, and fatality is concerning. As noted in prior studies, QT/QTc prolongation can induce early after depolarizations, provoke Torsades de Pointes, or lead to ventricular fibrillation, which can potentially cause sudden cardiac death. As such, QT/QTc prolongation appears to provide a plausible explanation for energy drink-induced ventricular arrhythmias. The relation between QTc and all-cause mortality, cardiac mortality, and sudden cardiac death has also been well-established in population studies (58). However, it is important to note that QTc prolongation does not always lead to Torsades de Pointes or ventricular arrhythmias, therefore additional markers for arrhythmia risk prediction should also be incorporated in future studies.

Supraventricular arrhythmia and myocardial ischemia were also frequently documented in case reports, but these abnormalities cannot necessarily be explained by the prolongation of the QT/QTc interval. A multitude of risk factors exist for supraventricular arrhythmias such as atrial fibrillation and atrial flutter, including high dose caffeine, alcohol, amphetamine salts, excessive sympathetic stimulation, and stress amongst others. That being the case, coingestion of ED with another substance may explain the supraventricular arrhythmias seen in published cases. Interestingly, of the 10 cases of supraventricular arrhythmia, 6 involved coingestion of a second substance (2 with alcohol, 2 with amphetamine salts, 1 with caffeine, and 1 with a caffeinated soda). In contrast, coingestants were only involved in 2 of the 11 cases of ventricular arrhythmia and 4 of the 14 cases with ST-segment changes.

Consumption of ED has also been linked to myocardial ischemia in multiple case reports (8, 9, 11, 16, 17, 19, 24, 26, 28, 30–32, 34, 35). Changes in platelet aggregation post-ED consumption has been suggested by some studies (59), warranting further exploration of this phenomenon as a pathway leading to coronary thrombosis.

Even though inconsistencies in outcome have been observed in clinical trials due to heterogeneity in study design and small sample size, one interesting trend was observed. There appears to be a dose-related relationship between ED consumption and QTc prolongation. In studies showing significant QTc prolongation with ED, the median volume of ED consumed was >800 ml, compared to <500 ml in studies showing a lack of association. The majority of reported cases also involved consumption of large amounts of ED. Limiting the volume of energy drinks acutely therefore should be encouraged.

Certain patient population may be at particular risk for developing ED-related adverse effects. The majority of the studies included young health volunteers; however, one study had a cohort of overweight or obese students (36) and another looked at patients with underlying LQTS (43). While QTc was not changed in the normal weight group, the overweight/obese group demonstrated significantly increased QTc at 60 min post-ED consumption (*p* = 0.006) (36). A meta-analysis conducted by Omran et al. seems to confirm the association between overweight/obesity and QTc prolongation (60), therefore this population may require further risk assessment. Although a significant change in QTc was not observed in the study conducted in patients with LQTS, 3 out of 24 patients in the study developed QTc increase of at least 50 ms after the ED consumption (43). Energy drink consumption also unmasked LQTS in 2 of the published cases (14, 25). Together, these data suggest that the consumption of ED should be limited in patients with underlying LQTS.

Whether it is an individual component in energy drinks, multiple ingredients, or their combination that may induce ECG abnormalities requires further investigation. Though most energy drinks contain caffeine, caffeine is generally recognized as safe at doses under 400 mg and seems unlikely to be the sole ingredient contributing to rhythm abnormalities in EDs. In a small study of 10 healthy volunteers, no ECG changes were evident 3 h post 400 mg of caffeine ingestion (61). Based on population studies, moderate caffeine consumption is also thought to be an unlikely cause of arrhythmias (62). As such, in the study by Fletcher et al. where ED was compared to a matching caffeine-only control, the QTc was 10 ms higher in the ED group when compared to the caffeine group (41). However, there was not a non-caffeinated-control group which would have allowed for a more ideal comparison. A recent study by Basrai et al. also evaluated the impact of a commercial ED against ingredients commonly included in ED. Comparator groups in the study included placebo, placebo with caffeine, placebo with taurine, placebo with glucuronolactone and placebo with caffeine and taurine (38). Despite the complex structure of the design, QTc prolongation was observed in the ED group while all other groups either had no change or QTc shortening. Lastly, in a study comparing ED, panax ginseng and a placebo control, panax ginseng did not appear to have an effect on ECG parameters while ED prolonged the QTc interval (50). It appears likely that the unique combination of ingredients in ED, as opposed to caffeine alone, may be responsible for inducing ED-associated ECG changes in clinical studies. Based on available information, taurine or panax ginseng alone does not appear to drive any ECG effects associated with EDs.

One important consideration in studies investigating the effect of ED and QTc is the formula used for QT interval correction. While Bazett's formula is most commonly used clinically, it has been known to overcorrect at higher heart rates and undercorrect at lower heart rates. Several other correction formulas exist, and some data suggest that the Fridericia and Framingham correction formulas have the best rate correction and significantly improved prediction of 30-day and 1-year mortality compared to the Bazett's correction formula (63). Most trials included in this review used the Bazett's correction formula, but future studies should consider reporting multiple correction formulas.

The impact of acute vs. chronic consumption also warrants further evaluation. In a number of case reports, subjects had been consuming energy beverages for an extended period of time before presenting with a cardiac abnormality (7, 14, 17, 19, 27, 28, 30, 32). Only 2 clinical trials evaluated the effects of chronic (1 week) ED consumption (49, 52). However, interpretation of these studies is difficult due to discordant results and study design limitations. With emerging data, multiple organizations started to raise a note of caution with ED consumption. In 2011, the American Academy of Pediatrics, an organization of 60,000 pediatricians recommended against kids and teens from drinking EDs (64). In 2013, the American Medical Association called for a ban in advertising EDs to children under 18 (65). Lithuania was the first country to enact a ban against the sale of EDs to minors (66). Aiming to safeguard the development of EDs in Europe, all European Union member states must now abide to specific provisions for the labeling of EDs under the Code of Practice for the Marketing and Labeling of Energy Drinks. Countries have either adopted specific rules on EDs (Germany, Switzerland) or provided principles for the composition of EDs through corresponding food guidelines (Austria) (67). Subsequently, the American Beverage Association also developed voluntary guidelines to label EDs for their age appropriateness in 2014 (68).

To our knowledge, this is the most comprehensive review as other reviews are only driven by case reports or have not captured the totality of available literature (69, 70). However, several limitations should be noted when interpreting our findings in this review. It is important to note that the majority of the studies were small, mainly studied in young healthy volunteers, assessed only the acute cardiovascular effects of ED consumptions and with some, lacked a control arm. This review also does not extend to soda type beverages which may also contribute to the total daily caffeine consumed by individuals (e.g., soft drinks typically contain between 20 and 70 mg of caffeine per 12 fluid ounces) (71).

## Conclusions

The preponderance of data suggests a significant correlation between ED and electrophysiological changes. While case reports have noted both ventricular and supraventricular arrhythmias, these were not evident in clinical trials. Large volume ED consumption appears to mildly prolong the QTc interval which may in part explain the mechanism behind some of the ventricular arrhythmias and associated deaths. Coingestion of other substances was seen in the majority cases of supraventricular arrhythmias. ST-segment changes and myocardial ischemia were also frequently noted in case reports. The exact mechanisms and the particular combination of ingredients behind these cardiac abnormalities require further evaluation. Until more information is available, energy drink use should be considered as part of the differential diagnosis in appropriate patients presenting with electrocardiographic changes. Appropriate labeling or warnings for consumers are also worthy of consideration by policy makers.

## Author's Note

Energy drinks are an increasingly utilized niche in the beverage market and are gaining popularity in recent years, especially among teenagers and young adults. An increasing number of cardiovascular adverse effects, emergency room visits, and deaths have been linked to the consumption of energy drinks. To our knowledge, this is the most comprehensive review assessing electrophysiological and ischemic adverse effects associated with energy drink consumption. Overall, 32 case reports and 19 clinical trials are included in this review, suggesting the need for thorough patient evaluation presenting with electrocardiographic changes. The exact mechanisms and the particular combination of ingredients behind energy drink-induced cardiac abnormalities require further evaluation. Until further information is available, certain patient populations should exercise caution and limit their energy drink consumption.

## Author Contributions

All authors listed have made a substantial, direct and intellectual contribution to the work, and approved it for publication.

## Conflict of Interest

SS has served as an expert witness in legal cases related to caffeinated energy drinks. The remaining authors declare that the research was conducted in the absence of any commercial or financial relationships that could be construed as a potential conflict of interest.
